# Adverse Hematological Effects of COVID-19 Vaccination and Pathomechanisms of Low Acquired Immunity in Patients with Hematological Malignancies

**DOI:** 10.3390/vaccines11030662

**Published:** 2023-03-15

**Authors:** Armand N. Yazdani, Nathaniel DeMarco, Parth Patel, Arian Abdi, Prathosh Velpuri, Devendra K. Agrawal, Vikrant Rai

**Affiliations:** Department of Translational Research, College of Osteopathic Medicine of the Pacific, Western University of Health Sciences, Pomona, CA 91766, USA

**Keywords:** COVID-19, vaccination, hematological malignancies, side effects, adverse events

## Abstract

The SARS-CoV-2 virus and the COVID-19 pandemic have spread across the world and severely impacted patients living with hematological conditions. Immunocompromised patients experience rapidly progressing symptoms following COVID-19 infection and are at high risk of death. In efforts to protect the vulnerable population, vaccination efforts have increased exponentially in the past 2 years. Although COVID-19 vaccination is safe and effective, mild to moderate side effects such as headache, fatigue, and soreness at the injection site have been reported. In addition, there are reports of rare side effects, including anaphylaxis, thrombosis with thrombocytopenia syndrome, Guillain-Barré Syndrome, myocarditis, and pericarditis after vaccination. Further, hematological abnormalities and a very low and transient response in patients with hematological conditions after vaccination raise concerns. The objective of this review is to first briefly discuss the hematological adverse effects associated with COVID-19 infection in general populations followed by critically analyzing the side effects and pathomechanisms of COVID-19 vaccination in immunocompromised patients with hematological and solid malignancies. We reviewed the published literature, with a focus on hematological abnormalities associated with COVID-19 infection followed by the hematological side effects of COVID-19 vaccination, and the mechanisms by which complications can occur. We extend this discussion to include the viability of vaccination efforts within immune-compromised patients. The primary aim is to provide clinicians with critical hematologic information on COVID-19 vaccination so that they can make informed decisions on how to protect their at-risk patients. The secondary goal is to clarify the adverse hematological effects associated with infection and vaccination within the general population to support continued vaccination within this group. There is a clear need to protect patients with hematological conditions from infection and modulate vaccine programs and procedures for these patients.

## 1. Introduction

The prevalence of SARS-CoV-2, or COVID-19, and its variants have increased tremendously in the last three years, resulting in more than 64 million cases and over 3 million deaths worldwide [1]. This has caused increased financial and socioeconomic hardship in households and hospital systems worldwide [2]. The virus is responsible for respiratory illnesses ranging from self-limited respiratory symptoms to severe pneumonia, including acute respiratory distress syndrome (ARDS), organ failure, and death [3]. Beyond the common symptoms of cough, fever, and loss of taste, COVID–19 is associated with a host of cardiovascular, neuropsychological, and hematological conditions, including thrombocytopenia, lymphopenia, leukocytosis, and disseminated intravascular coagulation (DIC) [2,4,5]. Patients suffering from hematological deficiencies are severely immunocompromised and are considered vulnerable subjects to severe disease progression. The risk of death in these patients from COVID-19 is twice as high as in subjects without a hematologic condition [5]. Unfortunately, data on the effectiveness of COVID-19 vaccination in this patient population is still limited [6].

Patients with underlying comorbidities, infected with COVID-19, have an increasingly rapid and severe progression of illness and mortality. Patients with hematological malignancies and infected with the virus, in particular, experience prolonged viral shedding and delayed seroconversion [5,7]. With these disease progressions in mind, there have been increased efforts to develop vaccines to protect vulnerable populations from contracting SARS-CoV-2, including the administration of over 13.07 billion COVID-19 vaccination doses globally to over 68.7% of the world population [8]. However, patients with hematological malignancies are at high risk for COVID-19 complications, with mortality rates exceeding 30% [9]. To date, there are four COVID-19 vaccines (Moderna, Pfizer-BioNTech, Novavax, and J&J/Janssen) approved for use in the United States, plus the AstraZeneca vaccine, yet there is limited data on the adverse effects in patients with hematological complications. Preliminary data suggest a poor seroconversion rate in vaccinated patients with hematological conditions [6]. Furthermore, there is no comprehensive review of efficacy data from vaccinated patients that is critical for improving policies [10]. Thus, it is crucial to understand the mechanistic aspects of vaccine-associated adverse events to improve outcomes. Here, we reviewed the potentially adverse hematological outcomes associated with COVID-19 infection and the mechanism by which complications may occur. In addition, we study the hematological pathomechanisms that occur following vaccination in the immunocompromised so that clinicians can make informed decisions on how to protect high-risk patients from infection. Finally, we dissect the adverse hematological complications associated with COVID-19 vaccination within the general population. 

## 2. Materials and Methods

**Figure 1:**
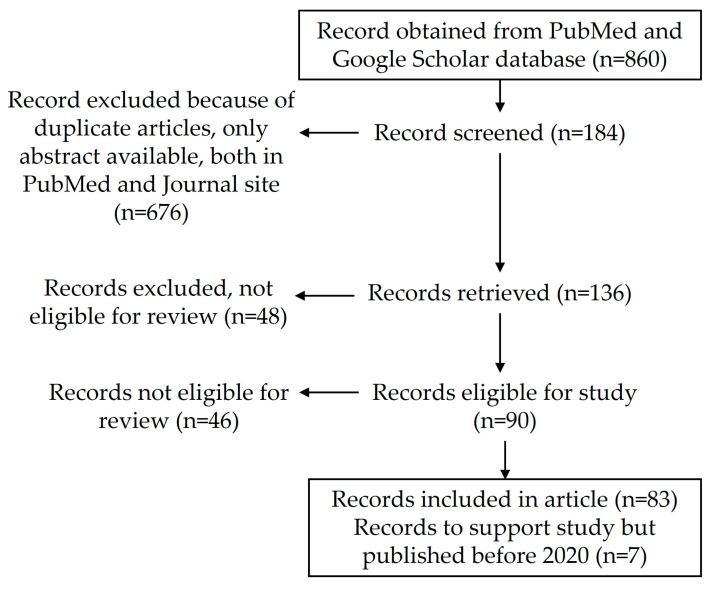
Article search and selection strategy for this review article.

A literature search was conducted using PubMed and Google Scholar to identify the background, mechanism, and treatment usage of COVID-19 vaccines. Articles were selected for inclusion based on keywords, alone or in combination, such as COVID-19, SARS-CoV-2, clinical findings, adverse effects, vaccines, vaccination, and hematological complications. A total of 860 articles were retrieved (COVID-19, hematological side effects, events = 818 articles and COVID-19, hematological malignancies, vaccination, side effects = 42 articles). The article selection to include in the review article was based on the article title and abstract, following which the full-text article was reviewed and included in the bibliography. The search was limited to peer-reviewed articles. The duplicate articles, only abstracts, non-English articles were excluded during the literature search following PRISMA guidelines (Figure 1). The findings from case reports, case series, and systemic reviews are summarized and discussed. 

## 3. Hematological Comorbidities and Clinical Outcomes Associated with COVID-19 Infection

### 3.1. Thrombocytopenia

The incidence of thrombocytopenia in patients with SARS-CoV-2 has varied widely. Rahma et al. reported the incidence to be 5–21% of all COVID-19 patients. The population mostly affected by thrombocytopenia have been mostly patients older than 50 and with severe disease [2]. A proposed mechanism for induced thrombocytopenia has been the infection of bone marrow cells that leads to abnormal hematopoiesis. The Coronavirus family can invade bone marrow cells through CD13 receptors in the host bone marrow. This mechanism has been shown in Human Coronavirus 229E, which is part of the coronavirus family. The thrombocytopenia caused by this virus is very similar to the one caused by SARS-CoV-2 [11].

### 3.2. Coagulation Abnormalities

COVID-19 patients as a population have elevated D dimer levels, and this indicates a higher likelihood of venous thromboembolism and disseminated intravascular coagulation (DIC) in COVID-19 patients [12]. The average thrombotic complication rate was measured to be 9.5% among COVID-19 patients, compared to the average of less than 1%, indicating a worse prognosis in coagulation abnormalities. Despite this, the mechanisms leading to COVID-19-induced coagulopathy are not well understood. Conway and his team showed that the overall premise could be categorized into three steps involving endothelial dysfunction or injury following a hyper-immune process ending with some form of hypercoagulability [13]. SARS-CoV-2 virus binds to angiotensin-converting enzyme (ACE)2 receptors, found on the surface of endothelial cells decreasing the expression of ACE2 [14]. A pre-existing or developing injury to the endothelial surface induces an acute inflammatory pathway consisting of the pain pathway involving bradykinin and angiotensin II (Ang II), which can be proliferated with a maladaptive hyperimmune state consisting of overproduction of cytokines, reactive oxygen species (ROS), and autoantibodies [15]. Complement activation and circulating antibodies can induce the production/misregulation of fibrin, von Willebrand factor (vWF), tissue factor, or more pathway compounds, or impair fibrinolysis leading to a state of hypercoagulability. 

Increased coagulation systematically can lead to infarcts and microthrombi, leading to multi-organ failure [16]. Spudich and colleagues found that the infarcts processed from misregulation of fibrinolysis can cross the blood-brain barrier. This strongly correlates to the increased likelihood of neurological symptoms found in patients with COVID-19 and elevated D dimer levels. Neurological manifestations in COVID-19 patients can encompass a wide spectrum of disorders, such as behavioral changes to ischemic strokes, resulting in increased in-hospital mortality [17]. Thus, patients with COVID-19, specifically those found with hypercoagulable states, are at an increased risk of neurological and cardiovascular manifestations. The risk of venous thrombosis is much higher in patients in critical conditions, specifically in immunocompromised and unvaccinated patient populations, with almost double the risk [18]. However, the data are relevant to those of the earlier strains. Despite elevated D-Dimer levels being a significant risk factor for developing venous thromboembolism (VTE)/deep vein thrombosis (DVT), it is not the definite factor, as it is used as an initial screening test as a marker of endogenous fibrinolysis and, thus, to rule out the blood clotting disorder.

### 3.3. Red Blood Cells and Hemoglobin 

COVID-19 is a disease that primarily affects the respiratory system. It is worth investigating the connections between COVID-19 and possible alterations in red blood cells (RBCs) and hemoglobin levels, structure, and function during infection. These potential alterations may affect the ability of the RBCs/hemoglobin to deliver oxygen to different areas of the body. In one study, it was found that during a COVID-19 infection, RBCs changed membrane homeostasis at the protein and lipid levels, which, in theory, improved the capacity of hemoglobin to off-load oxygen [19]. This is consistent with the idea that the body would want to adapt to COVID-19-induced hypoxia, thus allowing for more efficient oxygen delivery. However, further changes in membrane homeostasis may result in a decreased ability of RBCs to respond to environmental variations in oxidative stress when traveling through the body. 

Another noteworthy alteration is the change in red blood cell distribution width (RDW). RDW is a measure of the heterogeneity of erythrocyte volumes, with an elevated RDW indicating a possible disorder/disease. During earlier studies involving SARS-CoV-2 infection, it was found that an elevated RDW at the time of hospital admission, as well as an increase in RDW during hospitalization, was associated with increased mortality risk in COVID-19 patients [20]. A subsequent study investigating the RDW of patients infected with the delta variant of SARS-CoV-2 showed an improvement in anemic conditions, and the RDW was markedly lower overall, with only 4.2% of patients showing abnormally high values [21]. While the Delta variant is known to have increased transmissibility, the decrease in RDW suggests a potentially milder disease, within the context of RBC alterations.

When looking at morphological abnormalities of RBCs, Marchi et al. showed 65% of patients in their study cohort had some degree of RBC abnormalities [22]. Of the morphological abnormalities, spiculated red blood cells were the most frequent. Additionally, patients with >10% RBC abnormalities had an increased mortality rate of 41.9% when compared with the group with <10% or no abnormalities (20.5% and 12.5%, respectively). While these findings point to significant clinical implications of morphological changes within RBCs of COVID-19 patients and an increased mortality risk, further investigation is warranted. 

## 4. Adverse Hematological Effects of COVID-19 Vaccination within the General Population

### 4.1. Vaccine-Induced Immune Thrombocytopenia 

As more of the population becomes vaccinated, cases of hematological side effects of vaccinations have become more apparent. One of the more dangerous side effects includes thrombocytopenia and thromboembolisms due to vaccination. A case of thromboembolism in a patient with preexisting thrombocytopenia was reported after vaccination with the Oxford-AstraZeneca vaccine [23]. Another systemic review of 286 patients who experienced any form of thromboembolism after vaccination revealed that a significant proportion of patients also experienced thrombocytopenia, elevated D-dimers, and antiplatelet 4 antibodies [24]. Of note is that these complications were after the Astra-Zeneca vaccine, indicating a possible link between the delivery method of the vaccine and thromboembolic events. A mechanistic link between the adenovirus delivery method of the Astra-Zeneca COVID-19 vaccine and elevated antiplatelet 4 antibodies has been proposed. It has been shown that interactions between the adenovirus vector and platelet factor 4 (PF4) result in anti-platelet factor 4 antibodies, which can activate platelets and culminate in vaccine-induced thrombotic thrombocytopenia [25,26]. Additional mechanisms regarding the manufacturing process itself have been proposed, with vaccine-induced immune thrombocytopenia (VITT) to do the AstraZeneca vaccine also being attributed to manufacturing byproducts [27]. This simply suggests that there is no single cause of VITT and that additional analysis of vaccine components and off-target interactions will be needed to implicate any component. This has caused fears of adverse vaccine side effects in many, but the European Medicine Agency has issued a statement saying that VITT is not higher in the vaccinated subjects compared to the general population, with only 1.13 cases of the adverse effect among 100,000 doses [28,29]. 

Additionally, the risk of thrombotic events in response to vaccination is related to predisposing risk factors. A meta-analysis investigating the adverse effects of adenoviral vector vaccination noted cardiovascular thrombosis following DVT/PE in patients [30]. Another study focused on immune thrombotic thrombocytic purpura (iTTP) that implicated low levels of ADAMTS13 (a disintegrin and metalloproteinase with a thrombospondin type 1 motif, member 13) activity as one of the major factors in patients who received vaccination leading to an adverse thrombotic event [31]. Numerous mechanisms for iTTP have been proposed, ranging from electrostatic interactions with platelets to platelet consumption; however, neither have been definitely implicated [32]. Although it is not conclusive that low levels of ADAMTS13 lead to vaccine-induced thrombotic events, it is a predisposing factor that must be considered. More serious complications of VITT, such as cerebrovascular incidents, have also been explored in hopes of reducing mortality. Out of 35 million ChAdOx1 vaccine recipients, 169 cases of CVT were reported with an excess event rate of 2.5 cases per 100,000 [33]. Given the severity of this side effect, further studies have been conducted with the Ad26. The COV2.S (Janssen) vaccine is also being implicated. Again, the studies point toward adenoviral vectors used in both vaccines as the major factor in these complications [34]. Ultimately, however, it must be emphasized that despite these side effects, the benefits of the vaccines far outweigh the negative side effects, and these side effects must not be taken out of context.

### 4.2. Premenstrual and Menstrual Changes

Vaccination has had varying side effects between women and men in the past, with the human papillomavirus (HPV) vaccine being a key example. Differences in male and female biology contribute to this dichotomy, specifically due to the complex menstrual cycle in women. COVID-19 vaccination has been shown to have some side effects resulting in alterations in this cycle. A cross-sectional study with 14,153 women observed mild menstrual and premenstrual changes regarding certain cycle characteristics, such as menstrual pain, bleeding, and shorter cycle length [35]. Period onset was also affected with 79 participants in a prospective study reporting later than normal period onset after vaccination, which rapidly returned to normal shortly after [36]. Another literature review aggregated data from both published and preprint studies to determine the most common premenstrual and menstrual side effects after COVID-19 vaccination. Again, increased bleeding and bleeding at irregular intervals throughout the cycle were the most common side effects reported [37]. The sampling of 4989 participants in the UK resulted in 20% of individuals reporting some form of menstrual disturbance following vaccination [36]. All these studies point to premenstrual and menstrual changes being minor but presenting side effects in women following COVID-19 vaccination. Studies have shown conflicting results, with some showing an increase in cycle length and others showing decreased cycle length, but a possible explanation has been proposed [35,38]. The menstrual phase during which the vaccine is administered has been shown to possibly explain the dichotomy of results. Vaccination during the early to middle portion of the cycle, the follicular phase, resulted in increased mean cycle length, while vaccination during the late portion of the cycle, the luteal phase, resulted in shorter cycle length [39]. The exact mechanisms underlying these side effects have not been adequately investigated. This warrants further study to ensure that female patients can confidently choose a life-saving vaccine without fear of developing menstrual abnormalities.

### 4.3. White Blood Cells

White blood cells (WBCs) are heavily involved in the body’s immune response, warranting an investigation of the effects of COVID-19 vaccination on WBC levels. WBCs is an umbrella term used for granulocytes as well as other components of the cell-mediated and adaptive immune response. Depending on the characteristics of the infiltrating pathogen, certain WBCs play more important roles than others, such as CD8+ T-cells in patients with viral infections [40,41]. 

Given the fact that vaccination acts by inducing a controlled immune response to increase the likelihood of recurring infection by the same pathogen, there may be dangerous alterations in WBC levels, causing more damage than benefit. A study of 139 patients who received the BioNTech/Pfizer vaccine showed that less than 5% of patients had mild/moderate granulocytopenia or leukocytopenia [42]. Additionally, leukopenia as a side effect was present in only 0.01% of patients in BioNTech/Pfizer phase IV clinical trials with a 6.25% death rate among those who had leukopenia (Pfizer Data). These studies indicate that while COVID-19 vaccination does have an adverse effect on WBCs, the effect is not significant and is rarely fatal. 

When investigating reactions to vaccinations, it is important to understand that individual leukocyte classes may be elevated while the overall WBC level remains normal. Eosinophilia is one of many conditions that may present with normal WBC levels. Although not present in most cases, case reports regarding hypersensitivity reactions due to COVID-19 vaccination have surfaced with altered eosinophil levels commonly being the culprit. A case report presented a patient who experienced cellulitis 12 days after receiving the second dose of the BNT162b2 mRNA vaccine presenting with normal WBC but hyper-eosinophilia [43]. Another case report was published regarding a patient with eosinophilic dermatosis who had elevated leukocytes and eosinophils after receiving the AstraZeneca COVID-19 vaccine [44]. Again, it is important to note that these are case reports rather than meta-analyses, and therefore present rare complications of the vaccine. As such, further research comprising these individual cases must be performed to fully evaluate whether COVID-19 vaccination can truly cause dangerous complications due to the modulation of WBCs and determine the underlying cellular mechanisms in the occurrence of these effects.

### 4.4. Cardiovascular and Hematological Complications 

The SARS-CoV-2 virus has a markedly detrimental effect on the cardiovascular system of patients. Over 31% of patients affected with COVID-19 develop thrombotic complications with pulmonary embolisms despite thromboprophylaxis. Furthermore, patients affected with the COVID-19 virus may go on to develop DVT and ischemia of cardiac tissues secondary to cytokine-mediated endothelial injury. A considerable number of patients affected by the virus experience elevated D-Dimer and troponin levels upon hospitalization with COVID-19 [45,46,47,48]. There is an indication that the patients with SARS-CoV-2 virus experience a much higher inflammatory response compared to infection with other viruses. This is substantiated by elevated inflammatory markers such as C-reactive protein, IL-6, and ferritin in COVID-19 patients [48,49]. The long-term impact of the COVID-19 virus on cardiovascular health is still being studied, but there is an indication that 78% of patients recovering from COVID-19 have residual cardiac involvement [48]. One 12-month long-term study noted that the risk of stroke, atrial fibrillation, myocarditis, ischemic cardiomyopathy, and heart failure was 2–4 times higher in patients with prior COVID-19 infection compared to those not infected at all [50]. This is likely due to the high inflammatory burden placed on cardiac myocytes through the course of infection, leaving the tissues vulnerable to further complications [48]. 

While there are many cardiovascular complications associated with the SARS-CoV-2 virus, the COVID-19 vaccine carries its own risks. Cardiovascular (CV), hemorrhagic, and thrombosis events have been reported following vaccination efforts, but these events are rare [51,52]. The most common clinical cardiac symptoms experienced following COVID-19 vaccination include tachycardia, shortness of breath, palpitations, chest pain, and hypertension [51]. Cardiac pathologies that have been reported in association with COVID-19 vaccination include myocardial infarction (MI), myocarditis, myopericarditis, ischemic heart disease, hypertension, acute coronary syndrome, arrhythmia, and cardiac arrest [53]. Myocarditis has been recognized as the most common coronavirus vaccine-related disease in young adults and adolescent males with reports indicating over 12.5 million cases in individuals aged 12–39 years [54]. Patients with vaccine-induced myocarditis admit feelings of chest pain and have elevated troponin, abnormal ECG with ST elevation, and irregular myocardium on MRI 2–3 days following the second vaccine dose [52,55,56]. Patients with vaccine-associated myocarditis experience higher left ventricular ejection fraction and better outcomes compared to other causes of myocarditis. Of note, at short-term follow-up (median 22 days), all patients with vaccine-associated myocarditis are asymptomatic with no adverse events; this is a stark contrast to the long-term cardiovascular event associated with a majority of unvaccinated COVID-19 patients [48,57]. The mechanism for COVID-19 vaccine-induced myocarditis likely involves molecular mimicry between the SARS-CoV-2 spike protein and self-antigens including α-myosin, causing dysregulation of activation of immune pathways and dysregulated cytokine expression [52,55]. Overall, the incidence of myocarditis is more than seven times higher in persons who are infected with the SARS-CoV-2 virus compared to those who receive the COVID-19 vaccine [47].

Following COVID-19 vaccination, cardiovascular (CV), hemorrhagic, and thrombosis events were reported. Cardiac events that were reported included myocardial infarction (MI), myocarditis, myopericarditis, and ischemic heart disease. Recent data reveal cardiovascular and hematological events in patients are closely associated with the reception of the Pfizer, Moderna, and AstraZeneca vaccines. Most CV and thrombosis events were reported within the first 30 days of the administration of the first dose of the vaccine, but some cases of hemophilia were reported after the second dose [58]. For instance, a study following 406 individuals receiving COVID-19 vaccinations revealed over 1000 adverse CV or hematological events with 45% of the events being thrombotic [59]. However, the association between the COVID-19 vaccines and these conditions has not been confirmed. Furthermore, some studies have suggested that these conditions coincided with the vaccine administration [59,60]. 

Age and gender may also play a role in CV-related insults from COVID-19 vaccines. Studies report a twice as high incidence of thrombotic events in the female population compared to males, especially in the AstraZeneca and Pfizer vaccines [61]. This increased incidence could be due to the prothrombotic effects of higher estrogen levels in women of childbearing age [62]. The male predominance of myocarditis may be related to sex hormone differences in immune response or an underdiagnosis of cardiac disease in women [55]. It has also been shown that there were more cases of cardiovascular and hematological complications in the 35–54 age group [59], with younger patients at a greater risk for cardiovascular complications following the second dose [53]. One study that looked at the rate of emergency medical service calls in Israel regarding MI and acute coronary syndromes (ACS) noted a 25% increase in the volume of the calls, which was strongly associated with the first and second doses of the mRNA vaccine rollout [63]. However, the authors of this study noted some limitations, such as not knowing the COVID-19 status of the patients in question. Another study noted the protective role of COVID-19 vaccines against MI, ACS, and myocarditis post-COVID-19 infection [47,64]. A study conducted by the CDC further illustrated that the risk of CV adverse events was significantly lower following COVID-19 vaccination compared to COVID-19 infection [65]. 

The AstraZeneca vaccines have a particular, dose-related association with both cardiovascular and hematological events. The first dose of the Oxford-AstraZeneca vaccine has been associated with acute myocardial infarction and pulmonary embolism in the second week of vaccination. The Pfizer-BioNTech and Moderna vaccine did not have an association with PE and acute MI [66]. In a study comparing 1013 events of CV and hematological complications, it was indicated that 73.7% of the incidences were associated with the AstraZeneca vaccine. The rate of myocarditis and pericarditis were higher with the mRNA vaccines while the rate of MI and ischemic heart disease were higher with the AstraZeneca vaccine [59]. One possible mechanism for this difference in cardiovascular injuries could be the presence of polyethylene glycol (PEG) in the mRNA vaccines. Although PEG has been considered to be inert, there have been cases that report hypersensitivity reactions to PEG [67]. Individuals with this hypersensitivity reaction could develop an inflammatory process that leads to cases of myocarditis and pericarditis [59].

Some systematic reviews have reported higher rates of venous thrombosis compared to arterial thrombosis post-COVID-19 vaccination. Among the venous thrombosis, cerebral venous thrombosis (CVT) and cerebral venous sinus thrombosis (CVST) had the highest rate of occurrence, having a rate of 34.6% compared to all the thrombotic events [59]. Since these events were usually seen accompanying thrombocytopenia, it is hypothesized that the mechanism is similar to Heparin Induced thrombocytopenia (HIT). This mechanism involves the complex formation of Heparin and platelet factor 4 (PF4). The host will then produce antibodies against this complex. This antibody-bound complex can then interact with the Fc𝛾RIIA receptor on platelets, leading to a hypercoagulable state and the formation of arterial and venous thrombosis. This mechanism has also been shown in patients without any history of heparin use or with other compounds, such as polyvinyl phosphonate [68]. With the reported risks noted above, there has been some vaccine hesitancy for individuals already burdened with cardiovascular disease. However, there is no evidence of increased disease progression after receiving two doses of either the Pfizer or CoronaVac vaccine [69]. 

## 5. Vaccination Efficacy in Patients with Hematological Malignancies

Hematological malignancies consist of a collection of conditions originating from the cells of the bone marrow and lymphatic system. They are broadly categorized into leukemias, lymphomas, and plasma cell neoplasms [70]. Furthermore, provocative mechanisms of hematological malignancies, including bone marrow expansion, osteolysis, lymph node enlargement, and mucositis, lead to deep visceral and somatic pain in patients [71]. As the findings from new studies are released on the effects of COVID-19 vaccines, there is increased skepticism as to whether there is any benefit of vaccination in these populations. Preliminary data already suggest a low seroconversion rate in vaccinated patients with hematological conditions compared to healthy patients; in other words, these patients can only produce a limited antibody response to the vaccine [6,9,72]. 

The BNT162b2 mRNA COVID-19 vaccine (Pfizer) consists of a highly purified single-stranded 5′-capped mRNA produced from corresponding DNA templates; the modified mRNA encodes for the viral S glycoprotein of SARS-CoV-2 to enable expression of the SARS-CoV-2 antigen. It has been touted as having more than 90% effectiveness in preventing COVID-19 and was the first COVID-19 vaccine approved by the FDA [2]. However, when the Pfizer vaccine was introduced to patients with hematological neoplasms, the risk ratio for COVID-19 infection, symptomatic disease, hospitalizations, severe COVID-19, and death was significantly higher compared to vaccinated-matched healthy controls. Furthermore, patients receiving treatment for their hematological condition are at higher risk [6]. These findings are compounded by preliminary data suggesting that patients with hematological malignancies fail to produce titers of anti-SARS-CoV-2 antibodies. Impaired postvaccination T-cell immune response has been reported as well in immunocompromised patients [6,73,74,75]. 

Quantitative analysis of antibody response in patients with hematological malignancies utilizes a SARS-CoV-2 IgG extinction coefficient to establish a signal/cutoff ratio. Data from patients with hematological malignancies reveal that less than half (46%) develop an antibody response to the Moderna or Pfizer vaccine. Patients with chronic lymphocytic leukemia (CLL) are at particular risk, as only 23% have any detectable antibodies in response to the vaccine [9]. While the mechanism for the lack of immunity post-vaccination is still unclear, there is evidence to suggest abnormalities in cellular immunity in these vulnerable populations [6]. 

While these results mainly pertain to the first and second dosages, there is evidence of a functional immune response in cancer patients receiving a third COVID-19 vaccine dose. Interestingly, patients with blood cancers can benefit even more from a fourth vaccine dose, even if they have no detectable response to the first three dosages, especially when considering the Omicron variants [72,76]. Both neutralizing antibody response and SARS-CoV-2 T cell responses are increased following a fourth COVID-19 dosage in patients with hematological malignancies. However, patients receiving B-cell-depleting therapies see no beneficial results from a fourth dose after 12 months [1,76].

### 5.1. Acute Myeloid Leukemia and Myelodysplastic Syndrome 

Acute myeloid leukemia (AML) is an adult disease with a median age of 64 years at the time of clinical presentation, which accounts for 30% of all adult leukemias. The rate of incidence has been slowly increasing in the past decade [70]. It is characterized by the infiltration of the bone marrow, blood, and other tissues by hematopoietic cells that are abnormally/poorly differentiated, and highly proliferative [77]. Myelodysplastic syndrome (MDS), on the other hand, is a group of cancers characterized by ineffective hematopoiesis, cytopenia, and a risk of progression to more severe AML. Additionally, MDS is a late-onset disease with an incidence rate of 55.4 per 100,000 people per year among the age group greater than or equal to 80 years [78]. Both AML and MDS represent serious conditions that may be further complicated by COVID-19 infection. For example, one study highlighting both lymphoproliferative (non-Hodgkin lymphoma, myeloma, and chronic lymphoid leukemia (CLL)) and myeloproliferative (AML and MDS) malignancies showed severe/critical presentations of COVID-19 (~60%), with about 18% requiring admission in the intensive care unit (ICU). Additionally, AML had a higher mortality risk when compared with lymphoproliferative diseases [79]. 

As AML has been shown to have a higher mortality risk with COVID-19 than other hematological malignancies, this highlights the importance of being able to prevent COVID-19 transmission in these patients. Of course, prevention of transmission within the context of vaccination has its own risks, as mentioned previously. In a study by Mori et al., [80] patients with myeloid malignancies, including both AML and MDS, were assessed 3 months after their second COVID-19 vaccine dose (BNT162b2 or mRNA-1273) and evaluated for anti-spike (S) SARS-CoV-2 antibodies. Among this patient population, seroconversion rates for AML and MDS were 94.7% and 100%, respectively. Additionally, it is worth mentioning that patients undergoing maintenance therapy for their malignancy had lower antibody titers than in those not undergoing maintenance therapy. These results showcase the excellent response to the COVID-19 vaccine in patients with either AML or MDS, especially when compared with previous studies in which the vaccine had a poor response in patients with lymphoid malignancies [81].

Within the context of MDS, previous studies have looked at the humoral and T-cell responses to specific COVID-19 vaccines, ChAdOx1 and BNT162b2. In this study, MDS patient serum was assessed for various markers to determine the serological response 2 weeks after vaccine administration. Overall, ChAdOx1-treated patients mounted a humoral and cellular immune response. However, this response was less potent than the response after BNT162b2 administration. Specifically, the serological response for those patients given the ChAdOx1 vaccine was 76.2%, while patients given the BNT162b2 vaccine had a 100% serological response [82]. These results suggest the need for specific vaccine treatments for patients with MDS. Additionally, this also highlights the importance of investigating the different responses that various vaccines may have within the context of a specific disease state.

### 5.2. Myeloproliferative Neoplasms and Syndromes (Essential Thrombocythemia, Polycythemia Vera, Myelofibrosis, Chronic Myeloid Leukemia)

Myeloproliferative neoplasms and syndromes comprise another serious subset of hematological diseases. Among this group are essential thrombocythemia (ET), polycythemia vera (PCV), myelofibrosis (MF), and chronic myeloid leukemia (CML). ET is characterized by marked thrombocytosis with a high frequency of thrombosis and a median age of diagnosis at 60 years [40,41]. PCV is a myeloproliferative disorder that involves a hematocrit greater than 16.5 g/dL/49% in men and 16 g/dL/48% in women with an accompanying JAK/STAT mutation [42]. Similarly, MF is another myeloproliferative neoplasm associated with the dysregulation of JAK/STAT signaling pathways. MF has a median age at diagnosis from 69 to 79 years. Finally, CML is another malignancy originating from the hematopoietic stem cells in which the maturation of myeloid cells is dysfunctional due to dysregulation of the oncoprotein BCR-ABL1 [43]. 

Past studies involving these blood cancers have shown a staggering <40% serological response rate after receiving a single dose of the BNT162b2 vaccine [44]. However, this specific study was limited in its scope of results as it did not include CML subjects in the cohort. Therefore, a subsequent study was performed in which patients with CML, ET, PCV, MF, and MDS were assessed for their serological response following the BNT162bs or ChAdOx1 vaccine. Patients with chronic myeloid blood cancers showed a 2-week post-vaccine serological response rate of 58% after a single dose of BNT162b2 or ChAdOx1 vaccines. Additionally, when looking at the specific subgroups, CML had the highest seroconversion of 75% [45]. 

As with the myeloproliferative disorders characterized previously, it is important to also highlight the vaccine response in patients undergoing specific treatments for their underlying hematological malignancies. In a study by Guglielmelli et al. [51], patients with PV, ET, and MF, who were also undergoing treatment with ruxolitinib, a JAK/STAT signaling inhibitor, were assessed. After vaccination, antibodies for anti-S IgG, anti-RBD IgG, and neutralizing antibodies were at 38.8%, 33.3%, and 33.3%, respectively. In contrast, a cohort of patients who were not taking ruxolitinib had 91.6%, 91.6%, and 58.3% response rates in the same respective antibodies [46]. This further highlights the caution with which patients with hematological malignancies must adopt even after vaccine administration. 

### 5.3. Chronic Lymphocytic Leukemia

Chronic lymphocytic leukemia (CLL) is one of the most common B-cell malignancies in Western countries. It is characterized by the accumulation of CD5+/CD19+ B lymphocytes that lack immuno-protective function, ultimately increasing the risk of infection [47]. These patients are particularly vulnerable to COVID-19 infection as case fatality rates in CLL patients with symptomatic COVID-19 are 30–33% [48]. Across all hematological malignancies, patients with CLL have the lowest antibody signals (23%) after receiving the Pfizer or Moderna vaccine, suggesting that these patients are unable to develop any antibodies following vaccination [9,48,49]. Spike-specific antibody response is detectable in as low as 34% of patients with CLL compared to 94% in healthy patients when administered the ChAdOx1 vaccine. The CLL patients also have an antibody response 104-fold lower than the healthy control [49]. When the two-dose BNT162b2 COVID-19 vaccine is administered, seropositivity is among the lowest of all hematologic malignancies (47%) [1]. Furthermore, patients undergoing treatment for their CLL have significantly fewer (23%) detectable antibodies compared to treatment-naïve patients [50]. Of note, the antibody responses do increase after the second dose of the vaccine in CLL patients and there is no difference in antibody levels acquired from the BNT162b2 or ChAdOx1 vaccines [49].

The mechanism for the poor immune response to COVID-19 vaccinations in CLL patients may be related to treatment with Ruxolitinib at the time of the vaccine. Ruxolitinib, a JAK2 inhibitor, is used to control cytokine release syndrome and COVID-related hyperinflammation; therefore, it may have a role in suppressing the immune response following COVID-19 vaccination [1]. A large proportion of CLL patients are also receiving the Bruton’s tyrosine kinase (BTK) inhibitor ibrutinib, a drug that blocks the B cell receptor pathway, impairing immune response to vaccination [47]. Patients should begin BTK inhibitor therapy after their COVID-19 vaccination [49]. Patients using BCL2 inhibitors are also at risk for seronegativity following vaccination, likely due to a systemic increase in apoptosis of B-cell following treatment, which lowers the immunity gained from vaccination [1]. Clinically, patients with CLL, especially if receiving treatment, should be closely monitored and serologically tested following COVID-19 vaccination. Protective efforts such as mask-wearing and social distancing are also encouraged as the immune response may be transient in these patients. Table 1 summarizes the studies on the efficacy of COVID-19 vaccines.

## 6. Vaccination Efficacy in Patients with Solid Malignancies 

Patients affected with cancer have a higher likelihood of being infected with COVID-19, similar to many immunosuppressed states [87]. This higher infection rate corresponds to higher mortality and morbidity among these populations, so there is a need to identify a therapeutical intervention that could promote a better prognosis for COVID-19. However, in comparison to patients with hematological malignancies, patients with cancer have higher seroconversion rates and higher immunogenic rates as well, indicating a possible step of degradation of overall health in this population. A study showed patients administered vaccination as soon as possible indicated higher seroconversion as well as not using immunosuppressive therapies. The study highlighted the balance between immunological modulators of therapy for malignancy and infection [87].

Another study showed that patients with cancer using active neoplastic agents and who were vaccinated with the BNT162b2 vaccine had significantly higher negative seroconversion rates [88]. However, cancer patients using non-chemotherapy interventions had similar rates to that of the general population. This study highlighted that vaccinated patients with cancer might not be fully protected against malignancies associated with COVID-19 and will need strong precautionary measures. Finally, a third study showed that third vaccination or booster shots can boost neutralizing antibodies among cancer patients to levels like that of the general population [76]. However, still, patients with hematological malignancies had significantly reduced neutralizing antibodies with corresponding higher infection and mortality rates. 

## 7. Conclusions

Within the general population, the adverse hematologic effects from contracting the COVID-19 virus appear to be much worse than the adverse effects of vaccination. The benefit of vaccination, however, is not supported in immune-compromised patients. The current COVID-19 vaccine modalities have poor effectiveness within patients with hematological conditions as the majority are non-responders. Findings from the studies highlight the importance of adherence to non-pharmacological interventions in patients with hematological conditions, particularly regarding antiviral vaccine therapies [9]. The variability in vaccine response shown in the different immunocompromised populations demonstrates a need for larger systematic reviews of COVID-19 vaccine adverse effects and outcomes. The evidence at present shows a nuanced picture of vaccine response and calls for consideration of a variant-specific response in determining which patients may benefit from vaccines and what pharmaceuticals should be discontinued before vaccination [1,76]. Clinicians caring for patients with these conditions should be aware of the possibility of a low vaccine response in patients with hematological conditions and adjust their treatment accordingly. There is a clear need to protect patients with hematological conditions from infection and modulate vaccine programs and procedures for these patients. 

## Figures and Tables

**Table 1:** A summary of the original research findings on the efficacy of the COVID-19 vaccine in patients with hematological conditions.

Aim [Reference #]	Sample	Findings
Utilization of metabolomics, proteomics, and lipidomics to study changes in RBC structure/function [19]	29 molecularly diagnosed COVID-19 patients	RBCs had increased levels of glycolytic intermediates and significantly altered lipid metabolism, which both suggest changes in membrane homeostasis. This also suggests increased oxygen off-loading, as well as decreased capacity to respond to oxidative stress
To investigate the association between mortality risk and elevated RDW at the time of hospital admission and during hospitalization [20]	1641 adults diagnosed with SARS-CoV-2 infection and admitted to 1 of 4 hospitals in Boston, MA	Elevated RDW at the time of hospital admission and increase in RDW during hospitalization was associated with increased mortality risk in COVID-19 patients
To investigate the association between elevated RDW and mortality risk within SARS-CoV-2 delta variant infections [21]	677 COVID-19 Delta variant patients admitted at various centers in China	There was a decrease in anemic conditions within this cohort, as well as a decrease in RDW overall, with only 4.2% of patients showing abnormally high RDW values
To investigate RBC morphological changes associated with COVID-19 [22]	116 adult patients affected by COVID-19 between April and December 2020	65% of patients were found to have morphological abnormalities in their RBCs. Additionally, a follow-up study showed increased mortality among patients with <10% RBC abnormalities
Test effectiveness of the BNT162b2 mRNA COVID-19 vaccine in patients with hematological neoplasms [6]	37,899 vaccinated patients matched to 32,516 unvaccinated controls	Vaccinated patients with hematological neoplasms suffer from COVID-19 outcomes more than vaccinated individuals with intact immune systems
Identify the efficacy of the COVID-19 vaccine in hematological malignancy patients [9]	67 hematology malignancy patients receiving two mRNA vaccine doses	46% did not produce antibodies, and patients with B-cell CLL were at particularly high risk
Assess the functional immune response to COVID vaccinations in patients with cancer [76]	80 blood cancer patients receiving a third and fourth dose of BNT162b2	Patients with blood cancers can benefit from a fourth COVID-19 vaccine dosage, even if they have an undetectable response to the first three dosages
Study the serological response following two doses BNT162b2 COVID-19 vaccine in hematologic malignancies patients [1]	315 patients with hematologic malignancies and 108 control patients receiving the BNT162b2 vaccine	Chronic lymphocytic leukemia patients had the lowest rate of seropositivity post-vaccination, followed by non-Hodgkins lymphoma and multiple myeloma
Study spike-specific antibody response following first and/or second dose of COVID-19 vaccination [83]	299 patients with CLL receiving BNT152b2 and ChAdOx1 COVID-19 vaccination	Spike-specific antibody response is detectable in 34% of patients with CLL compared to 94% in healthy donors. Antibody titers 104 fold lower in the CLL group
Understand serologic response to mRNA vaccination in CLL patients [84]	44 CLL patients receiving two doses of mRNA (BNT162b2 or mRNA-1273) vaccine	Half of CLL patients vaccinated for COVID develop any detectable anti-SARS-CoV-2 S1/S2 antibodies
Assess risk factors associated with lymphoproliferative and myeloproliferative malignancies within the context of COVID-19 infection [79]	1084 patients with lymphoproliferative malignancy and 684 patients with myeloproliferative malignancy	Patients with hematological malignancies were at higher risk for severe/critical COVID-19, but the highest mortality was observed in acute myeloid leukemia and myelodysplastic syndrome patients
Assess serological response in AML and MDS patients 3 months after vaccination [80]	69 patients with AML or MDS receiving 2nd mRNA-based COVID-19 vaccination	Patients with MDS showed a significantly lower antibody titer than that in healthy controls (HCs) or AML patients; AML patients had a comparable serological response when compared with HCs, but lower in patients undergoing maintenance therapy
Assess the response (serological and T-cell) of MDS patients to different COVID-19 vaccine types [82]	38 patients with MDS receiving either BNT162b2 mRNA or ChAdOx1 nCoV-19 vaccines	Overall T-cell response to the BNT162b2 and ChAdOx1 vaccines were 71.4% and 70.6%, respectively. Overall serological responses to the BNT162b2 and ChAdOx1 vaccines were 100% and 76.2%, respectively
Assess the serological response of myeloid cancer patients to different COVID-19 vaccine types [85]	60 myeloid cancer (CML, ET, MF, PCV, MDS) patients receiving either BNT162b2 or ChAdOx1 nCoV-19 vaccines	After a single vaccination dose, only 58% of patients with chronic myeloid blood cancers showed seroconversion, with the highest rate of seroconversion in CML patients
Assess the serological response of MPN patients undergoing ruxolitinib treatment to the COVID-19 vaccine [86]	30 patients with PV, ET, and MF receiving Ruxolitinib treatment and a first dose COVID-19 mRNA vaccine	38.8%, 33.3%, and 33.3% of patients undergoing Ruxolitinib treatment had a serological response for the anti-S IgG, anti-RBD IgG, and neutralizing antibodies, respectively.

## Data Availability

Not applicable as this is a review article.
